# Supplementary material for: Hepatoprotection of a Standardized Extract of Cultured Lentinula edodes Mycelia against Liver Injury Induced by Ischemia-Reperfusion and Partial Hepatectomy
Source: Nutrients. 2024 Jan 14;16(2):256. doi: 10.3390/nu16020256 (PMC10820669; doi:10.3390/nu16020256)
Supplement: Supplementary file 1 [file nutrients-16-00256-s001.zip › nutrients-2785683-supplementary.pdf]

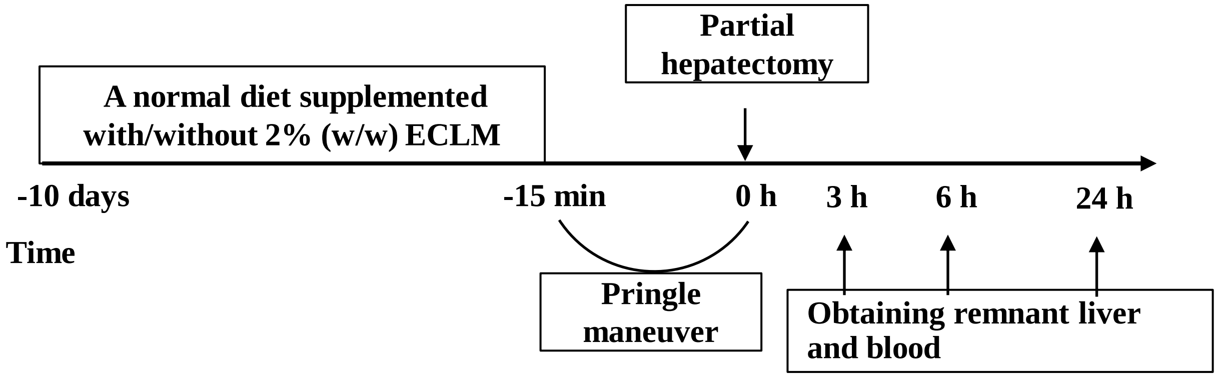

Sampling Experiment

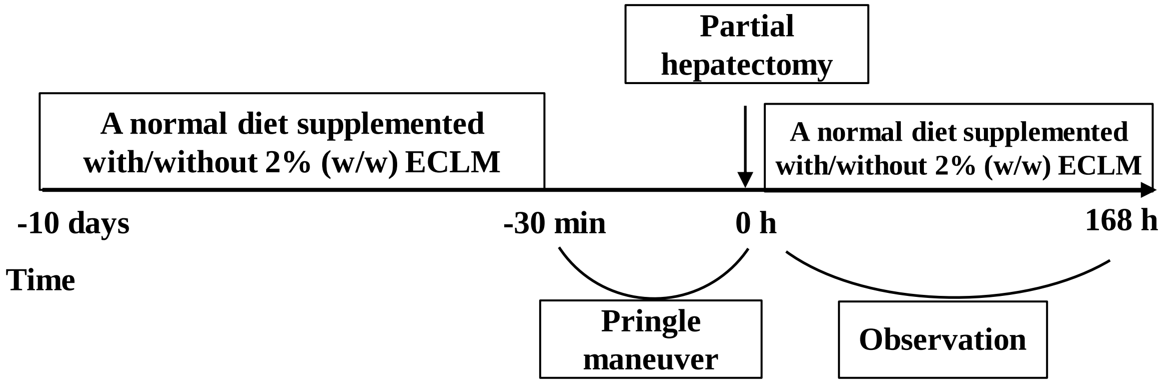

Survival Experiment

Figure S1

Supplementary Table S1 Primers used for RT-PCR to detect mRNA

	Sequence (5′ → 3′)	Direction	Length (bp)
CXCL-1	GCCAAGCCACAGGGGCGCCCGT	Forward	231
	ACTTGGGGACACCCTTTAGCATC	Reverse	
TNF-α	TCCCAACAAGGAGGAGAAGTTCC	Forward	275
	GGCAGCCTTGTCCCTTGAAGAGA	Reverse	
IL-10	GCAGGACTTTAAGGGTTACTTGG	Forward	245
	CCTTTGTCTTGGAGCTTATTAAA	Reverse	
MCL1	CTGGGGCAGGATTGTGACTC	Forward	169
	CACAAACCCATCCCAGCCTCTTTG	Reverse	
HGF	GCTACACTGGATTGATCAACGC	Forward	169
	CCATAATCTCCCTCACAAGGTC	Reverse	
iNOS	CCAACCTGCAGGTCTTCGATG	Forward	257
	GTCGATGCACAACTGGGTGAAC	Reverse	
eNOS	CAGCCCTAAGACCTATGTGCAAG	Forward	219
	TAGCGTTGCTGATCCCGCAGCA	Reverse	
EF	TCTGGTTGGAATGGTGACAACATGC	Forward	335
	CCAGGAAGAGCTTCACTCAAAGCTT	Reverse	

RT-PCR reverse transcription-polymerase chain reaction, *bp* base pairs, *EF* elongation factor 1α

Supplementary Table S2 Histological index in the liver of the HIRI+PH-treated rats

	Average ± SD	***P* value** HIRI+PH vs HIRI+PH and ECLM
Suzuki score		
3 h		
HIRI+PH	4.0 ± 0.0	
HIRI+PH and ECLM	5.3 ± 3.8	1.000
6 h		
HIRI+PH	8.0 ± 2.6	
HIRI+PH and ECLM	1.8 ± 0.4	0.010*
24 h		
HIRI+PH	3.2 ± 0.7	
HIRI+PH and ECLM	4.8 ± 2.9	0.879
TUNEL-positive cells/mm^2		
6 h		
HIRI+PH	145.9 ± 45.9	
HIRI+PH and ECLM	22.3 ± 14.0	0.004**
MPO-positive cells/mm^2		
6 h		
HIRI+PH	79.2 ± 29.5	
HIRI+PH and ECLM	22.3 ± 11.2	0.020*
Ki-67-positive nuclei (%)		
24 h		
HIRI+PH	1.9 ± 0.6	
HIRI+PH and ECLM	4.3 ± 1.1	0.007**

* $p < 0.05$ and ** $p < 0.01$ versus HIRI+PH alone.
